# Cardiac biomarkers response under angiotensin receptor–neprilysin inhibitor: a sub-analysis of the NATRIUM-HF study

**DOI:** 10.1093/eschf/xvag075

**Published:** 2026-03-16

**Authors:** Jolie Bruno, Aziz Daghmouri, Malha Sadoune, Romane Lafontaine, Alexis Nguyen, Christopher Edwards, Beth Davison, Gad Cotter, Koji Takagi, Christos Varounis, Priyanka Morishetty, Feriel Azibani, Elisabeth Masson, Camille Chenevier-Gobeaux, Alexandre Mebazaa, Benjamin Deniau

**Affiliations:** INSERM UMR-S 942, Cardiovascular Markers in Stress Condition (MASCOT), 2 rue Ambroise Paré, Paris 75010, France; Université de Paris Cité, 45 Rue des Saints-Pères, Paris 75006, France; Department of Anesthesiology, Critical Care and Burn Unit, University Hospitals Saint-Louis et Lariboisière, AP-HP, 1 Av. Claude Vellefaux, Paris 75010, France; Department of Cardiology, Inselspital, Bern University Hospital, University of Bern, Freiburgstrasse 20, Bern 3010, Switzerland; Department of Anesthesiology and Critical Care, Groupment Hospitalier de Territoire Grand Paris Nord-Est, Hôpital André Grégoire, Montreuil, France; INSERM UMR-S 942, Cardiovascular Markers in Stress Condition (MASCOT), 2 rue Ambroise Paré, Paris 75010, France; Université de Paris Cité, 45 Rue des Saints-Pères, Paris 75006, France; INSERM UMR-S 942, Cardiovascular Markers in Stress Condition (MASCOT), 2 rue Ambroise Paré, Paris 75010, France; Université de Paris Cité, 45 Rue des Saints-Pères, Paris 75006, France; INSERM UMR-S 942, Cardiovascular Markers in Stress Condition (MASCOT), 2 rue Ambroise Paré, Paris 75010, France; Université de Paris Cité, 45 Rue des Saints-Pères, Paris 75006, France; Momentum Research, Inc., Durham, NC,USA; Momentum Research, Inc., Durham, NC,USA; Momentum Research, Inc., Durham, NC,USA; Momentum Research, Inc., Durham, NC,USA; Abbott Laboratories, Chicago, IL, USA; Momentum Research, Inc., Durham, NC,USA; INSERM UMR-S 942, Cardiovascular Markers in Stress Condition (MASCOT), 2 rue Ambroise Paré, Paris 75010, France; Université de Paris Cité, 45 Rue des Saints-Pères, Paris 75006, France; Biochemistry Laboratory, University Hospitals Saint-Louis et Lariboisière, AP-HP, Paris, France; Biochemistry Laboratory, Cochin Hospital, AP-HP, Paris, France; INSERM UMR-S 942, Cardiovascular Markers in Stress Condition (MASCOT), 2 rue Ambroise Paré, Paris 75010, France; Université de Paris Cité, 45 Rue des Saints-Pères, Paris 75006, France; Department of Anesthesiology, Critical Care and Burn Unit, University Hospitals Saint-Louis et Lariboisière, AP-HP, 1 Av. Claude Vellefaux, Paris 75010, France; Université de Paris Cité, 45 Rue des Saints-Pères, Paris 75006, France; Department of Anesthesiology, Critical Care and Burn Unit, University Hospitals Saint-Louis et Lariboisière, AP-HP, 1 Av. Claude Vellefaux, Paris 75010, France; INSERM PARCC UMR 970, Paris, France

**Keywords:** Natriuretic peptides, Neprilysin, Angiotensin receptor-neprilysin inhibitor, Heart failure, Volume overload

## Abstract

**Introduction:**

Natriuretic peptides (NPs) are central to the diagnostic and therapeutic management of heart failure (HF), yet their short-term dynamics under sacubitril/valsartan (S/V) therapy and during acute volume changes remain incompletely characterized. We aimed to assess changes in circulating biomarkers and response to standardized acute intravascular volume expansion and diuretic treatment before and after S/V initiation.

**Methods:**

We studied 229 ambulatory patients with HF with reduced ejection fraction receiving guideline-directed medical therapy who initiated S/V. Patients were evaluated at three outpatient visits: before S/V initiation and after 2 and 3 months of treatment. At each visit, participants underwent a standardized 9-hour protocol including volume infusion followed by diuretic administration. BNP, NT-proBNP, MR-proANP, and neprilysin activity were measured serially alongside clinical assessment and natriuresis.

**Results:**

Across visits, initiation of S/V was associated with lower concentrations of BNP (−8%, *P* = .009) and NT-proBNP (−35%, *P* < .001). During the acute protocol, both BNP and NT-proBNP increased significantly over time (timepoint effect *P* < .001), with parallel trajectories before and after S/V initiation and no visit-by-time interaction (*P* = .17 for BNP; *P* = .95 for NT-proBNP). Despite marked natriuresis and improvement in clinical signs following diuretic administration, BNP and NT-proBNP continued to rise during the 9-hour observation period.

**Conclusion:**

In a controlled acute volume overload setting, BNP and NT-proBNP provide comparable information, but their short-term dynamics lag behind clinical decongestion. Routine serial NP measurements at very short time intervals are unlikely to add incremental clinical value in the early phase of acute HF.

## Graphical Abstract

**Graphical Abstract:**
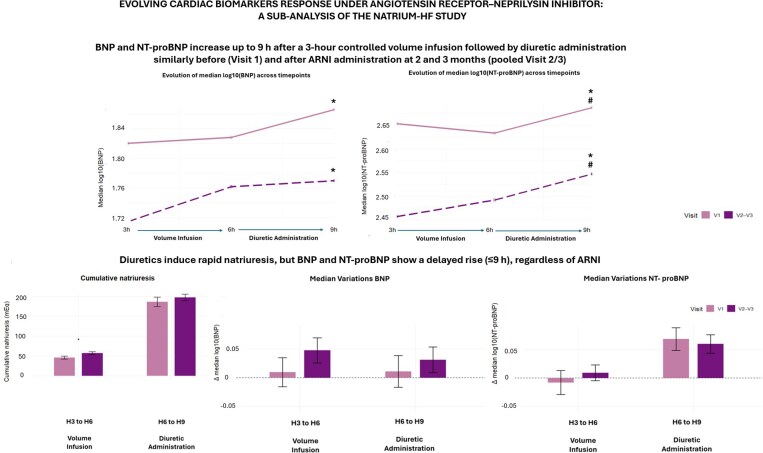
In a controlled model of acute fluid overload, both BNP and NT-proBNP increased significantly over 9 hours following a 3-hour volume infusion (H3–H6) and subsequent diuretic administration (H6–H9), both before and after ARNI therapy. Natriuresis increased rapidly after diuretic treatment, whereas circulating natriuretic peptides continued to rise. This temporal dissociation limits the clinical value of very early serial natriuretic peptide measurements.

## Introduction

Natriuretic peptides (NPs) play a central role in heart failure (HF) pathophysiology, reflecting myocardial wall stress and serving as indispensable biomarkers for diagnosis, risk stratification, and therapeutic monitoring.^1,2^ Neprilysin (NEP), a metalloprotease, constitutes a key regulatory node in the NPs system by catalysing the enzymatic degradation of several vasoactive substrates. Sacubitril/valsartan (S/V), an angiotensin receptor-neprilysin inhibitor (ARNI), has demonstrated significant clinical and prognostic benefits in HF patients^3–7^ by enhancing the NPs system while reducing maladaptive neurohormonal activation.^2,8–10^ Unlike N-terminal pro–B-type natriuretic peptide (NT-proBNP), which is not a NEP substrate, atrial natriuretic peptide (ANP) to a greater extent, and B-type natriuretic peptide (BNP) to a lesser extent, are directly influenced by NEP inhibition, and their interpretation during ARNI therapy is further complicated by assay-related and biological variability.^11–17^ While NT-proBNP consistently declines in the absence of congestion after treatment initiation,^8,17–20^ BNP has shown more heterogeneous short-term patterns, remaining stable or demonstrating modest increases or decreases within the first 8–10 weeks of treatment,^17,21^ thereby raising uncertainties regarding its utility for monitoring short-term treatment response. Despite this variability in the early phase, BNP retains its prognostic significance over the long term.^18,21,22^ Data on BNP and NT-proBNP kinetics during acute congestion are still scarce,^23^ and the extent to which ARNI therapy influences their behaviour in an acute setting remains unclear.

The NATriuretic Response to expansion and dIUretics in huMans with Heart Failure (NATRIUM-HF) study, from which the present sub-analysis is derived, applied a model of controlled acute volume overload in stable patients with heart failure with reduced ejection fraction (HFrEF).^24^ In the primary study, changes in diuresis, natriuresis, and congestion were assessed in response to standardized fluid and sodium loading, both before and after initiation of S/V therapy. The main findings demonstrated that, at 2 and 3 months after treatment initiation, patients exhibited a significantly enhanced renal response to acute volume expansion, characterized by increased diuresis and natriuresis, along with a concomitant reduction in symptoms of congestion. Moreover, natriuretic response to intravenous (iv) loop diuretics was also augmented after ARNI initiation.

By combining acute fluid loading and subsequent diuretic administration, this design provides an opportunity to assess the dynamics of cardiac biomarkers in response to rapid changes in haemodynamic load before and after S/V initiation.

## Methods

NATRIUM-HF (NCT04235062), a multicentre, non-randomized, pre–post intervention trial, aimed to assess the renal response to intravascular fluid, sodium loading, and diuretic treatment following the administration of S/V in euvolemic patients with HFrEF. The study design has been published elsewhere.^24^ Briefly, the study enrolled 229 ambulatory patients with stable HFrEF who remained symptomatic despite HF optimal treatment with angiotensin-converting enzyme inhibitor (ACEi) or angiotensin receptor blocker (ARB), beta-blocker (BB), and a mineralocorticoid receptor antagonist (MRA) for over 3 months, making S/V indicated.^1^ Patients were monitored across three outpatient visits: before initiation (Visit 1, V1), at two (Visit 2, V2), and three (Visit 3, V3) months post-initiation of ARNI.

During each visit, patients underwent an evaluation divided into three-hour phases. The phases included: a rest phase (0–3 h), a ‘volume infusion’ phase (3–6 h), during which 1 L of Ringer solution was infused intravenously over the first 2 h (from 3 to 5 h), followed by 1-hour without any further intervention, and a ‘diuretic administration’ phase (6–9 h) at the beginning of which iv furosemide 40 mg was administered. At every phase, vital signs, clinical assessments (dyspnoea score, jugular venous pulse, peripheral oedema score, pulmonary rales), and urine sample were obtained in a time lapse of three hours. Blood samples were collected at baseline, at the end of the rest and load phases, and every hour during the diuretic phase. The investigation conforms with the principles outlined in the *Declaration of Helsinki.* The study received approval from the relevant regulatory and ethics committees, and all participants provided written informed consent.

### Biomarker measurements

For each visit, blood samples were obtained at 0, 3, 6, 7, 8, and 9 hours after inclusion (hereafter referred to as H0, H3, H6, H7, H8, and H9, respectively). Blood was collected on EDTA-containing tubes and immediately centrifuged. Plasma samples were subsequently aliquoted, frozen locally at −80°C, and shipped to Inserm UMR-S 942 (Paris, France) for centralized biomarker analyses of BNP, NT-proBNP, mid-regional pro–atrial natriuretic peptide (MR-proANP), and NEP activity. BNP was measured using the Abbott chemiluminescent microparticle immunoassay (Architect i1000/i2000SR), with a measuring range of 10–5000 ng/L and intra-laboratory coefficients of variation (CVs) < 7%. NT-proBNP was quantified using the Roche electrochemiluminescent assay (cobas®8000, Roche Diagnostics, Meylan, France), with a measuring range of 5–35 000 ng/L and CVs <5%. MR-proANP was measured using the Kryptor assay (Thermo Fisher Scientific B·R·A·H·M·S, Courtaboeuf, France) with an intra-assay and inter-assay CVs of ≤ 5% and ≤ 6.5%, respectively, and a measuring range of 2.1 pmol/L–10 000 pmol/L. NEP activity was assessed by spectrofluorometry, as previously described.^25^ MR-proANP and NEP activity were measured only at the timepoint (H) 0 of each visit, for technical constraints.

### Study endpoint

Building on the previously reported findings, this analysis assesses changes in circulating biomarkers and their pathophysiological response to acute intravascular volume expansion and subsequent diuretic treatment both before and after S/V initiation. These analyses were pre-specified as secondary endpoints and had not been included in the primary report.^24^

### Statistical analyses

Continuous variables are reported as mean (standard deviation) or median (interquartile range), as appropriate. Normality of distribution was assessed using the Shapiro–Wilk test.

Cross-sectional distributions of cardiac biomarkers at H0 were displayed using violin plots (with median and IQR overlays) for each visit. The interval from H0 to H3 was pre-specified as a stabilization phase, during which biomarker kinetics can be non-stationary and heterogeneous. To ensure comparability, longitudinal mixed-effects analyses and within-visit comparisons were conducted from H3 onward, specifically at H3, H6, and H9. V2 and V3 were plotted to allow simple comparison pre- and post-S/V treatment. Longitudinal changes in BNP and NT-proBNP were visualized as the median of log10-transformed concentrations per timepoints and by visit; interquartile ranges (IQRs) are provided separately in the Supplementary Figures to enhance data visualization. Linear mixed-effects models were fitted with fixed effects for timepoint, visit, and their interaction, and a subject-specific random intercept. Estimated marginal means (EMMs) were derived on the log scale and back-transformed to geometric means, for clearer interpretation, with 95% confidence intervals (CIs). Model-based geometric means with 95% CIs (back-transformed EMMs) are shown in the Supplements. Post-hoc pairwise contrasts were adjusted with Bonferroni correction.

We plotted bar charts, with 95% t-based confidence intervals, of cumulative natriuresis during the H6–H9 phase alongside median change in log10(BNP) and log10(NT-proBNP), stratified by visit. Between-group differences in these mean changes (V1 vs. V2/V3) were assessed using Welch’s two-sample *t*-test (two-sided).

For BNP, NT-proBNP, and MR-proANP, concentrations below the lower limit of quantification (LLOQ) were retained and set to half the LLOQ (0.5×LLOQ). Negative NEP values were deemed analytically implausible (likely due to background subtraction and curve inversion near the detection limit) and treated as non-quantifiable. Accordingly, these values were replaced by the smallest observed positive NEP at that timepoint to preserve time-specific analytical variability. Participants whose biomarkers were all <LLOQ at all three timepoints were excluded *a priori*. The remaining missing observations were also excluded. Overall missingness was low: BNP, 94/3731 (2.5%); NT-proBNP, 96/3731 (2.6%); MR-proANP, 19/626 (3.0%); NEP, 0 (0%). All analyses were performed using R statistical software (version 4.4.3), and a two-sided *P*-value <.05 was considered statistically significant.

## Results

### Study population

The baseline characteristics of the study population have been described previously.^24^ Briefly, the mean age was 64 years, and the majority were men (74%). Most patients presented in NYHA functional class II (94%), with ischaemic aetiology as the predominant cause of HF (83%) and a mean left ventricular ejection fraction (LVEF) of 34%. Medical therapy was largely optimized: 97% of patients were on renin–angiotensin system inhibitors (RASI), 97% on mineralocorticoid receptor antagonists (MRA), and 100% on beta-blockers, with stable proportions across study visits. Approximately 40% of patients were on chronic loop diuretic therapy, with a mean furosemide-equivalent dose of 30 mg/day.

### Biomarkers at baseline

**Figure 1:**
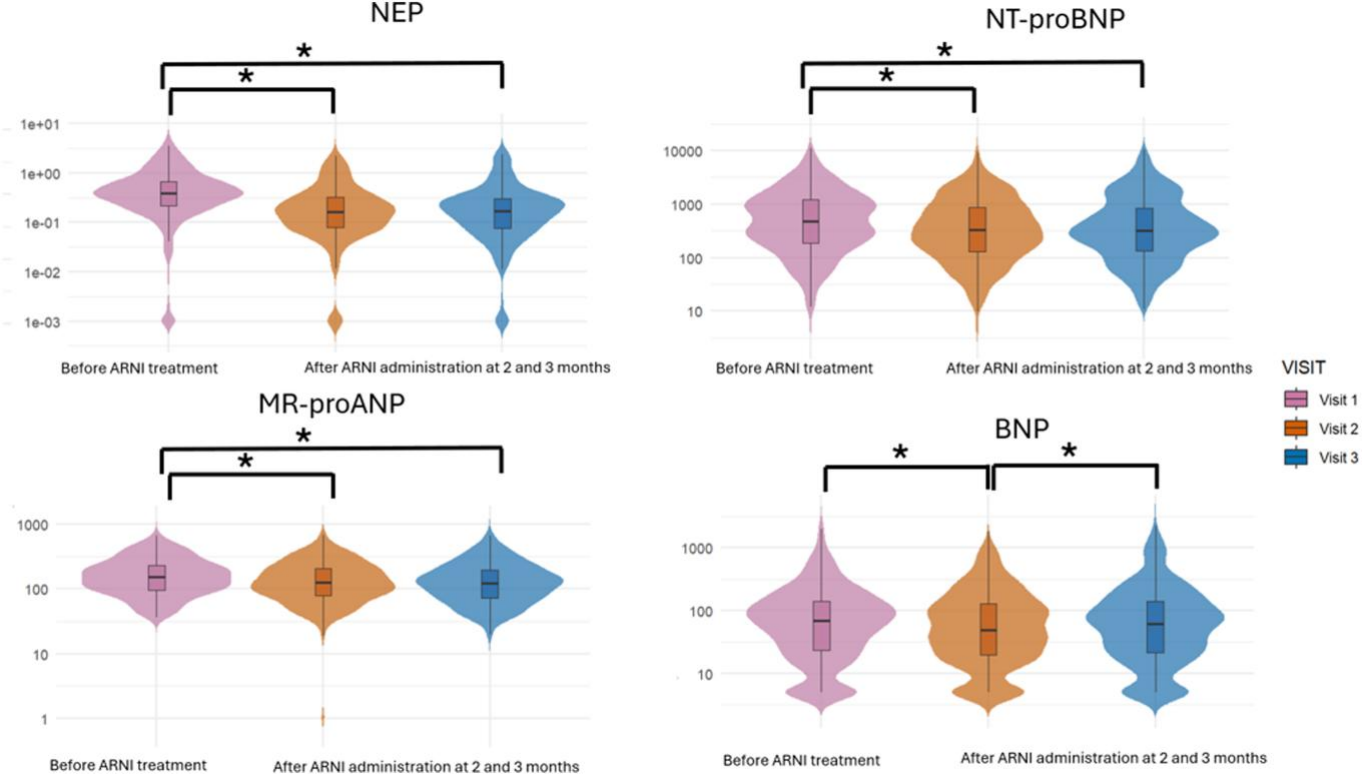
Temporal trends of biomarkers at timepoint 0 across visits. Violin plots (with overlaid boxplots) show the distributions of log10-transformed biomarker levels at Visit 1, Visit 2, and Visit 3: neprilysin activity (NEP), NT-proBNP, MR-proANP, and BNP. The violin width reflects kernel density; the central line denotes the median, and the box indicates the interquartile range. Asterisks denote pairwise differences between visits that were statistically significant (Bonferroni-adjusted) in the repeated-measures mixed-effects analysis. Sample sizes by visit (V1/V2/V3): NEP, *n* = 207/203/199; NT-proBNP, *n* = 210/201/199; MR-proANP, *n* = 215/190/202; BNP, *n* = 207/203/199

At baseline (H0), NEP activity was significantly lower at V2 and V3 compared with V1, while no difference was observed between V2 and V3. A similar trend was observed for NT-proBNP and MR-proANP (all *P* < .001). In contrast, BNP was significantly lower at V2 compared with V1 (*P* = .001), whereas no difference was observed between V1 and V3. BNP at V3 was significantly higher than at V2 (*P* = .011). Cross-sectional distributions at H0 are displayed in violin plots with median and interquartile range overlays (*Figure 1*).

### Temporal evolution of BNP and NT-proBNP

**Figure 2:**
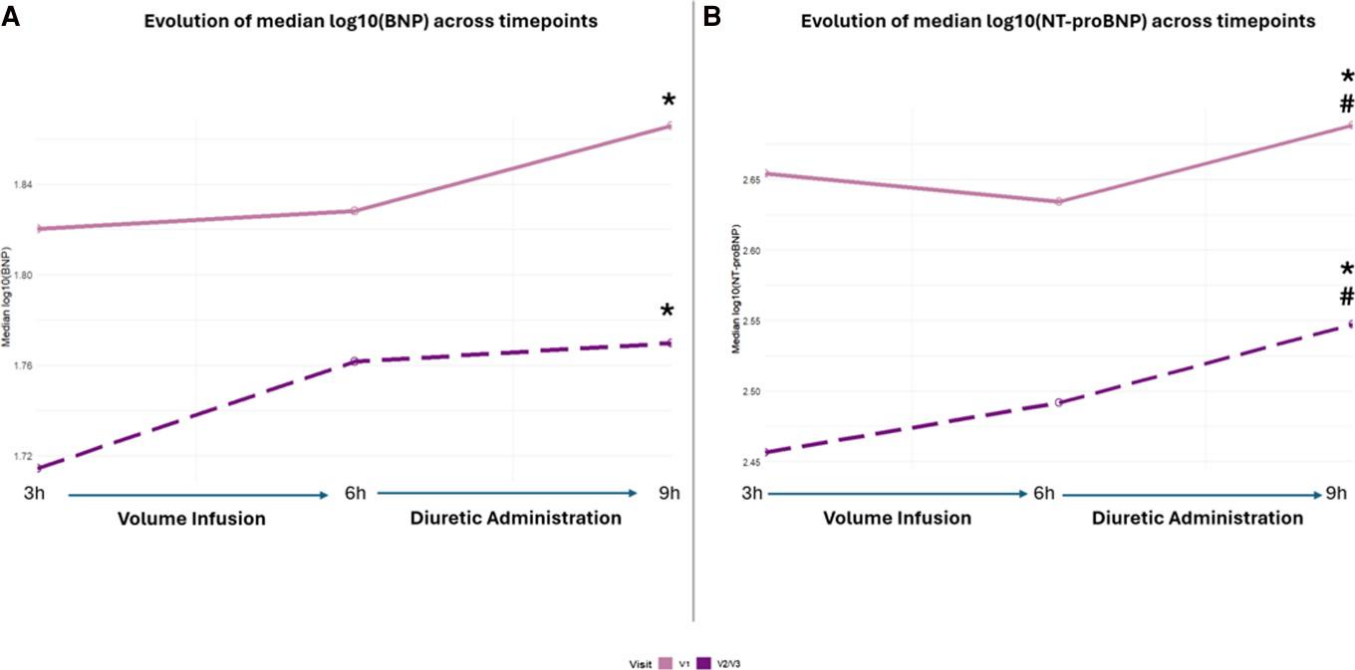
Median temporal changes in BNP and NT-proBNP across visits. Panels *A* and *B* display the median change in BNP and NT-proBNP, respectively, at timepoint 3 (H3), 6 (H6), and 9 (H9) for each visit (colors/linetypes identify Visit 1 and plotted Visit 2/Visit 3). The initial stabilization interval H0–H3 is not shown. The H3–H6 window corresponds to volume infusion, and H6–H9 to diuretic administration. Asterisk denotes statistically significant differences (Bonferroni-adjusted) between timepoints H3-H9, hash between H6 and H9. Sample sizes (*n*) by visit and timepoint: BNP—Visit 1: 208/207/208; Visit 2/Visit 3: 209/208/208. NT-proBNP—Visit 1: T3/T6/T9 = 208/208/208; Visit 2/Visit3: 209/208/208

The temporal evolution of BNP and NT-proBNP absolute concentrations (non–log-transformed) across visits is summarized in Supplementary Table S1. Median within-visit values for timepoint are illustrated in *Figure 2*.

For BNP (*Figure 2A*), repeated-measures ANOVA on log10-transformed values showed a significant effect of timepoint (*P* = .0046) and visit (*P* = .009), with no significant timepoint-by-visit interaction (*P* = .17), indicating a similar temporal trajectory before and after initiation of ARNI therapy. Post-hoc analyses demonstrated a significant increase in BNP concentrations at H9 compared with H3 (+11%, *P* = .003), whereas no significant differences were observed between H3 and H6 (volume infusion phase) or between H6 and H9 (diuretic administration phase). In line with the absence of a significant timepoint-by-visit interaction, the change of BNP during the diuretic phase (H6–H9) did not differ between V1 and pooled V2/3 (*P* = .61). Across visits, BNP concentrations were lower at pooled V2/3 compared to V1 (−8%, *P* = .009).

NT-proBNP showed a comparable trajectory (*Figure 2B*). Concentrations increased significantly over time (*P* < .001), with no significant interaction between timepoint and visit (*P* = .95). Post-hoc analyses confirmed that NT-proBNP did not differ between H3 and H6, but was significantly higher at H9 compared with both H3 (+16%, *P* < .0001) and H6 (+16%, *P* < .001). Similarly, analysis of NT-proBNP trajectories during the diuretic phase (H6–H9) revealed no difference between V1 and pooled V2/3 (*P* = .72). Across all visits, NT-proBNP concentrations were higher at V1 compared with pooled V2/3 (+35%, *P* < .001). Sample sizes for each timepoint are provided in the figures.

Supplementary Figure S2 illustrates median values with interquartile ranges, while Supplementary Figure S3 presents geometric means with corresponding 95% CIs.

To complement absolute trajectories and account for baseline differences between visits, biomarker changes were additionally analysed as relative variation (Δ) from H3. With this approach, BNP exhibited a differential response according to visit, with a greater percentage increase from H3 to H6 and from H3 to H9 at pooled V2/3 compared with V1 (*P* = .016 and *P* = .007, respectively). In contrast, relative changes in NT-proBNP (ΔNT-proBNP) did not differ between visits over the same intervals (from H3 to H6, *P* = .13; from H3 to H9, *P* = .58).

### Relationship of biomarker changes with natriuresis

**Figure 3:**
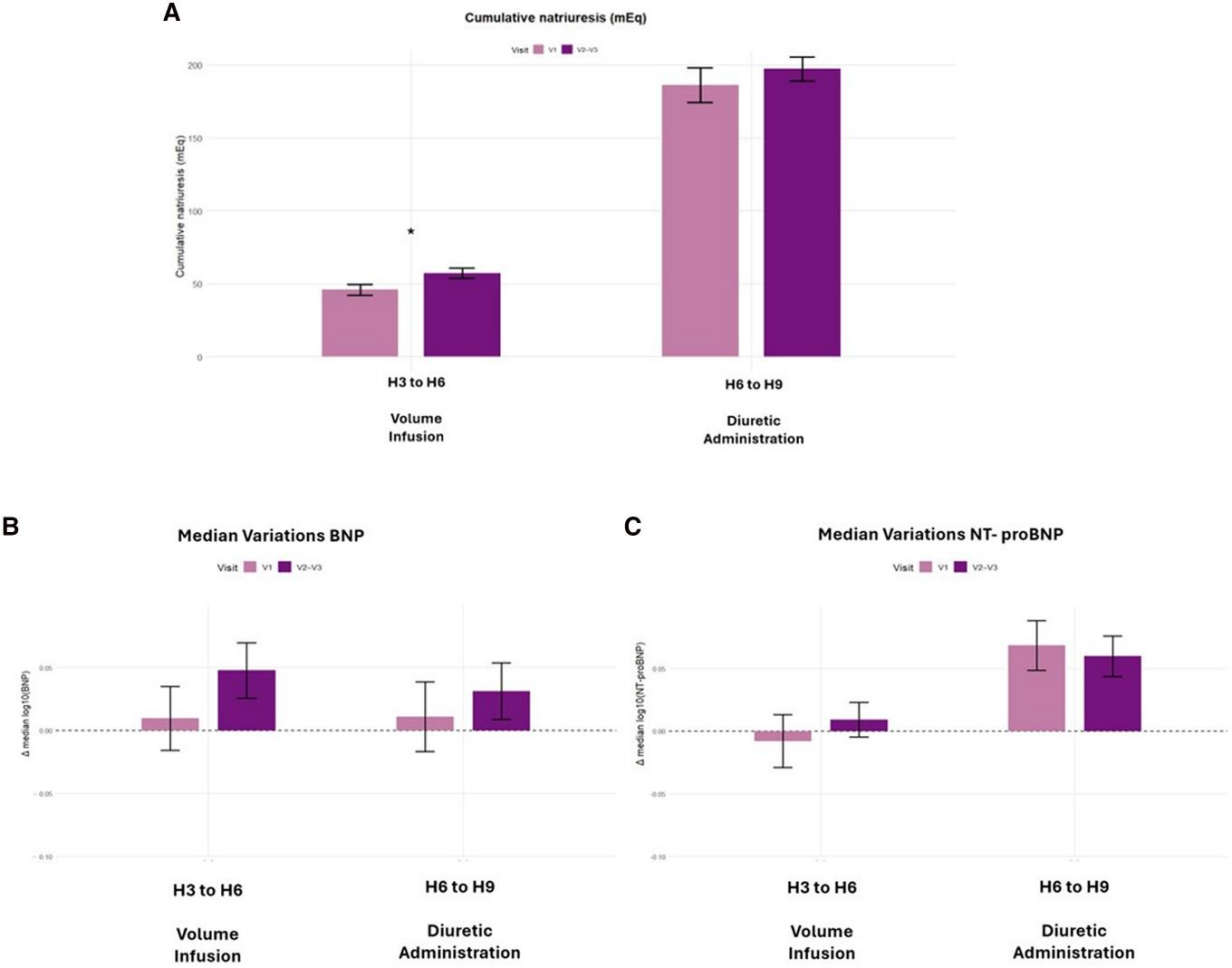
Changes in natriuresis and natriuretic peptides across the volume infusion (from timepoint 3 to timepoint 6; H3 to H6) and diuretic administration (from timepoint 6 to timepoint 9; H6 to H9) phases, comparing visit 1 (V1) with pooled visits 2–3 (V2/3). (*A*) Cumulative natriuresis. (*B*) BNP: change in the median log10(BNP). (*C*) NT-proBNP: change in the median log10(NT-proBNP). Positive bars indicate increases from the start to the end of each interval; negative bars indicate decreases. Error bars indicate 95% confidence intervals. Main observation: natriuresis markedly increased during the diuretic phase (H6 to H9), whereas BNP and NT-proBNP continue to rise over the same interval. Natriuresis in V1 compared with V2/3 was statistically significant (*) for H3–H6 (*P* < .001), whereas all other changes were not significantly different

*Figure 3* illustrates cumulative natriuresis and NPs dynamics across the volume infusion (H3–H6) and the diuretic administration (H6–H9) phases, comparing V1 with pooled V2/V3. As shown in panel a, cumulative natriuresis, derived from data previously reported in the primary NATRIUM-HF study,^24^ markedly increased during the duretic phase. Cumulative natriuresis during the volume infusion phase (H3–H6) differed significantly between V1 and pooled V2/3 (*P* < .001).

Panels 3b and 3c depict changes in BNP and NT-proBNP, respectively. BNP increased during the volume infusion phase and continued to rise during the diuretic administration phase, despite effective decongestion, as supported by enhanced natriuresis (panel a) and improvement in clinical signs reported in the primary study. NT-proBNP showed a similar trajectory, with sustained increases through H9. The magnitude of change in either BNP or NT-proBNP did not differ between V1 (before ARNI) and pooled V2/3 (under ARNI therapy).

When patients were stratified according to cumulative natriuretic response during the diuretic administration phase (high vs. low, based on the median value), changes in BNP and NT-proBNP did not differ between groups at either visit (all *P* = ns) (Supplementary Figure S4).

## Discussion

This study provides two complementary perspectives: the temporal short-term dynamics of natriuretic peptides (NPs) and NEP activity in patients with chronic, stable HF receiving S/V, and the acute behaviour of the BNP and NT-proBNP in a model of rapid volume overload simulating acute HF (AHF).

The observed decline in NT-proBNP during the early phase of follow-up after S/V initiation is consistent with previous reports^8,17–20^ and has been linked to reverse cardiac remodelling^26–28^ and, at least in part, to the diuretic effect of S/V alone^24^ and in combination with background GDMT and diuretics.^29^ In addition to unloading-related mechanisms, part of the apparent NT-proBNP reduction may reflect increased glycosylation, which can interfere with antibody recognition and lower measured concentrations without necessarily indicating a change in peptide production.^17^ In contrast, BNP concentrations showed variable fluctuations, most evident within the first 2–3 months,^17,21^ underscoring the complexity of using BNP alone to monitor short-term HF trajectories, due to multiple competing mechanisms, that can influence circulating concentrations.^2,12,17,18^ These fluctuations likely reflect the interplay between reduced wall stress, altered peptide metabolism under NEP inhibition, and dynamic changes in synthesis and clearance. This framework plausibly explains the lower BNP levels observed at V2, followed by a partial rebound towards baseline at V3.^9^

For MR-proANP, our data align with two previous studies reporting a reduction following ARNI initiation.^11,18^ Although NEP inhibition increases circulating bioactive ANP through enhanced synthesis, corin activation, and reduced degradation,^9,30^ MR-proANP represents a stable mid-regional fragment reflecting cardiac wall stress rather than active hormone concentrations and is not a substrate of NEP. Accordingly, its decline under ARNI therapy is physiologically plausible and likely mirrors reduced atrial wall stress and reverse remodelling, underscoring the distinct biological information conveyed by MR-proANP compared with ANP.

Regarding NEP activity, we observed a rapid and sustained inhibition by sacubitril, consistent with prior reports demonstrating a dose-dependent effect, with the most pronounced decline between no treatment and half-dose exposure and only modest additional reductions at full dose.^15^

Concerning acute NPs dynamics, this study demonstrates that although S/V treatment was associated with lower absolute concentrations of both BNP and NT-proBNP, the short-term biomarker response to standardized volume loading and unloading remained preserved, as reflected by parallel temporal trajectories before and after treatment. When responses were expressed as relative changes from rest, BNP, but not NT-proBNP, showed a larger proportional increase after ARNI initiation. This finding reflects normalization to lower post-ARNI BNP concentrations related to NEP inhibition, rather than an amplified acute haemodynamic response.

Our findings suggest in addiction that effective decongestion and clinical improvement occurred immediately during acute decongestive therapy, whereas changes in circulating NPs were delayed by more than 3 hours. BNP and NT-proBNP continued to rise despite marked natriuresis and improvement in clinical signs, and their changes were not associated with the magnitude of the natriuretic response. The delay decline in NPs may reflect, at least in part, the intrinsic biology of NPs, which are continuously synthesized rather than stored. Thus, the temporal gap between decongestion and biomarker response likely mirrors the physiological kinetics of peptide production rather than persistent congestion or ineffective therapy.

From a clinical perspective, our findings support two key concepts. First, BNP and NT-proBNP appear to provide comparable information in the acute setting, as both biomarkers exhibited parallel temporal patterns in response to standardized haemodynamic stress. This observation is particularly relevant given that, in several countries and healthcare systems, BNP remains the only NP assay routinely available. Second, serial NPs measurements over very short time intervals of only few hours appear to offer limited incremental clinical information, whereas assessment at presentation and before discharge remains most informative. A reduction of at least 30% in NPs concentrations has been associated with effective decongestion and improved post-discharge outcomes.^2^ Further research is warranted to clarify the biological mechanisms underlying the delayed NPs responses during acute decongestive therapy.

This study has several limitations. The sample size may limit the generalizability of the findings to a broader population. The duration of follow-up may not be sufficient to fully assess the long-term effects of S/V on NPs regulation. Additionally, the absence of invasive haemodynamic monitoring prevents direct correlation between biomarker changes and cardiac filling pressures. The lack of a control group limits the ability to isolate the specific effects of S/V from other concurrent HF therapies. Moreover, variations in clinical management (other than the study schema) could act as confounders. It is also important to acknowledge that our experimental model reflects a state of acute volume overload rather than the full pathophysiological spectrum of HF. As such, not all mechanisms involved in the disease may be faithfully reproduced. Finally, interpreting NPs trends remains challenging due to the influence of various extra-cardiac factors.

In conclusion, our study highlights that clinical signs of congestion in AHF may improve more rapidly than measurable changes in circulating NPs in the very early phase of a controlled acute volume overload setting. BNP and NT-proBNP provided comparable information in this context. These findings argue against routine serial NPs measurements at very short time intervals and emphasize the need for a more integrated approach to support therapeutic decisions-making in AHF.

## Supplementary Material

xvag075_Supplementary_Data

## Declarations

### Data Availability

No data were generated or analysed for this manuscript.

### Pre-registered Clinical Trial Number

None supplied.
