# Supplementary material for: Cardiac biomarkers response under angiotensin receptor–neprilysin inhibitor: a sub-analysis of the NATRIUM-HF study
Source: ESC Heart Fail. 2026 Mar 16;13(2):xvag075. doi: 10.1093/eschf/xvag075 (PMC13042280; doi:10.1093/eschf/xvag075)
Supplement: xvag075_Supplementary_Data [file xvag075_supplementary_data.docx]

**Supplementary data**

**Table 1. Biomarker levels across visits and timepoints.**Values are reported as medians with interquartile ranges (IQR). Biomarkers include BNP (pg/mL), NT-proBNP (pg/mL), MR-proANP (pmol/L), and neprilysin (NEP, nmol/μL/min).

|  | **T0** | **T3** | **T6** | **T7** | **T8** | **T9** |
| --- | --- | --- | --- | --- | --- | --- |
| **BNP, Visit 1** | n=207; 71 (24–139) | n=208; 66 (25–139) | n=207; 67 (23–148) | n=207; 66 (22–142) | n=209; 67 (24–133) | n=208; 73 (26–138) |
| **BNP, Visit 2** | n=203; 46 (19–123) | n=202; 46 (17–127) | n=201; 55 (20–122) | n=200; 53 (18–121) | n=198; 55 (21–132) | n=201; 47 (20–154) |
| **BNP, Visit 3** | n=199; 63 (24–130) | n=197; 56 (20–130) | n=197; 62 (19–141) | n=199; 64 (21–149) | n=197; 66 (22–157) | n=197; 67 (25–151) |
| **NTproBNP, Visit 1** | n=210;  462 (174–1125) | n=208;  451 (177–1161) | n=208;  431 (172–1155) | n=207;  457 (183–1235) | n=208;  515 (192–1288) | n=208;  488 (199–1302) |
| **NTproBNP, Visit 2** | n=201;  288 (117–785) | n=202;  287 (117–766) | n=200;  321 (124–817) | n=201;  344 (128–850) | n=197;  330 (132–892) | n=199;  353 (145–960) |
| **NTproBNP, Visit 3** | n=199;  309 (118–840) | n=199;  286 (130–777) | n=197;  301 (132–821) | n=197;  322 (139–800) | n=196;  346 (139–907) | n=198;  353 (140–888) |

**Figure 1.**

**1a) Evolution of Median log10(BNP) across Timepoint with IQR.**

**
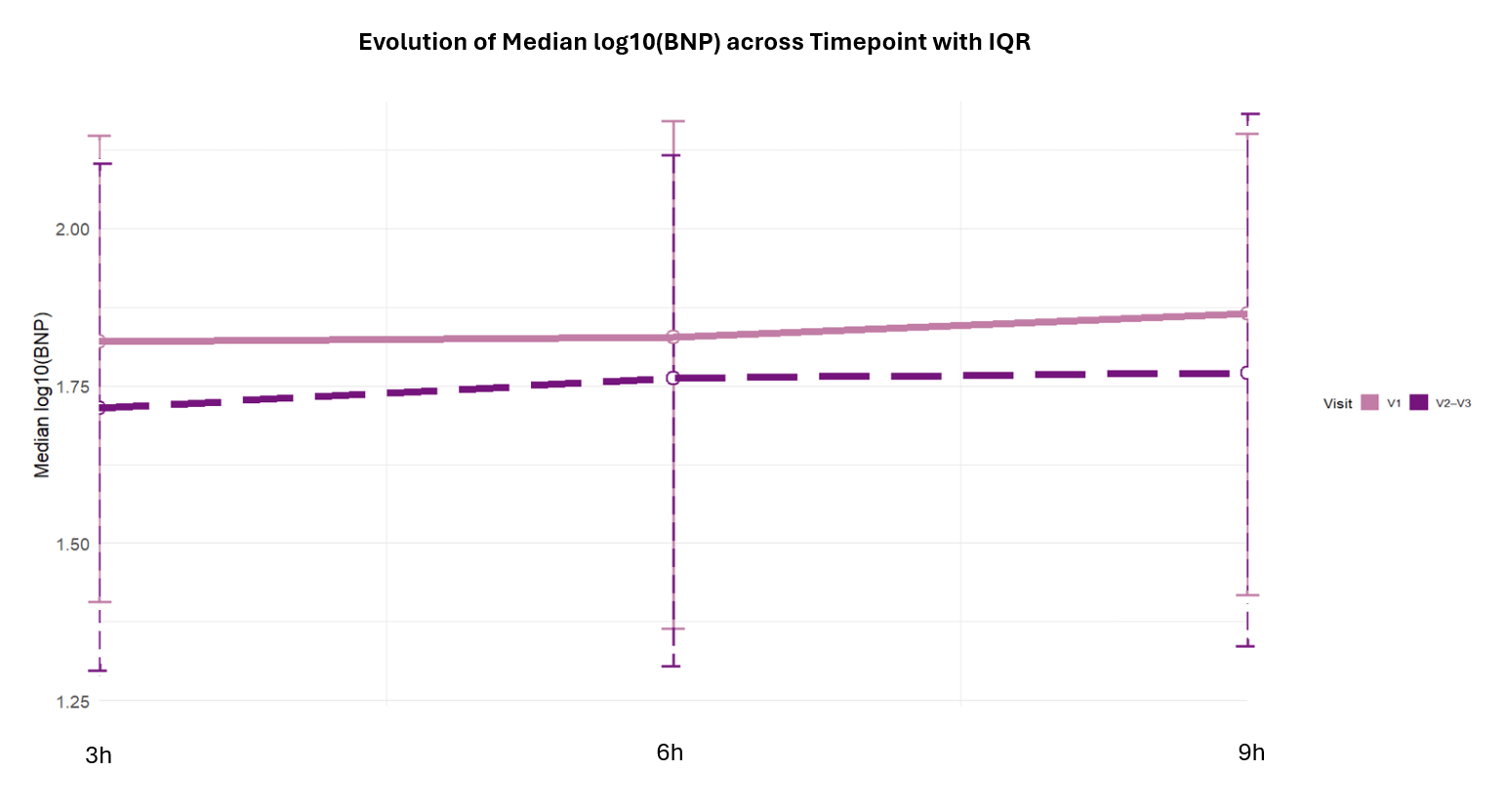
**

**1b) Evolution of Median log10(NT-proBNP) across Timepoint with IQR.**

**
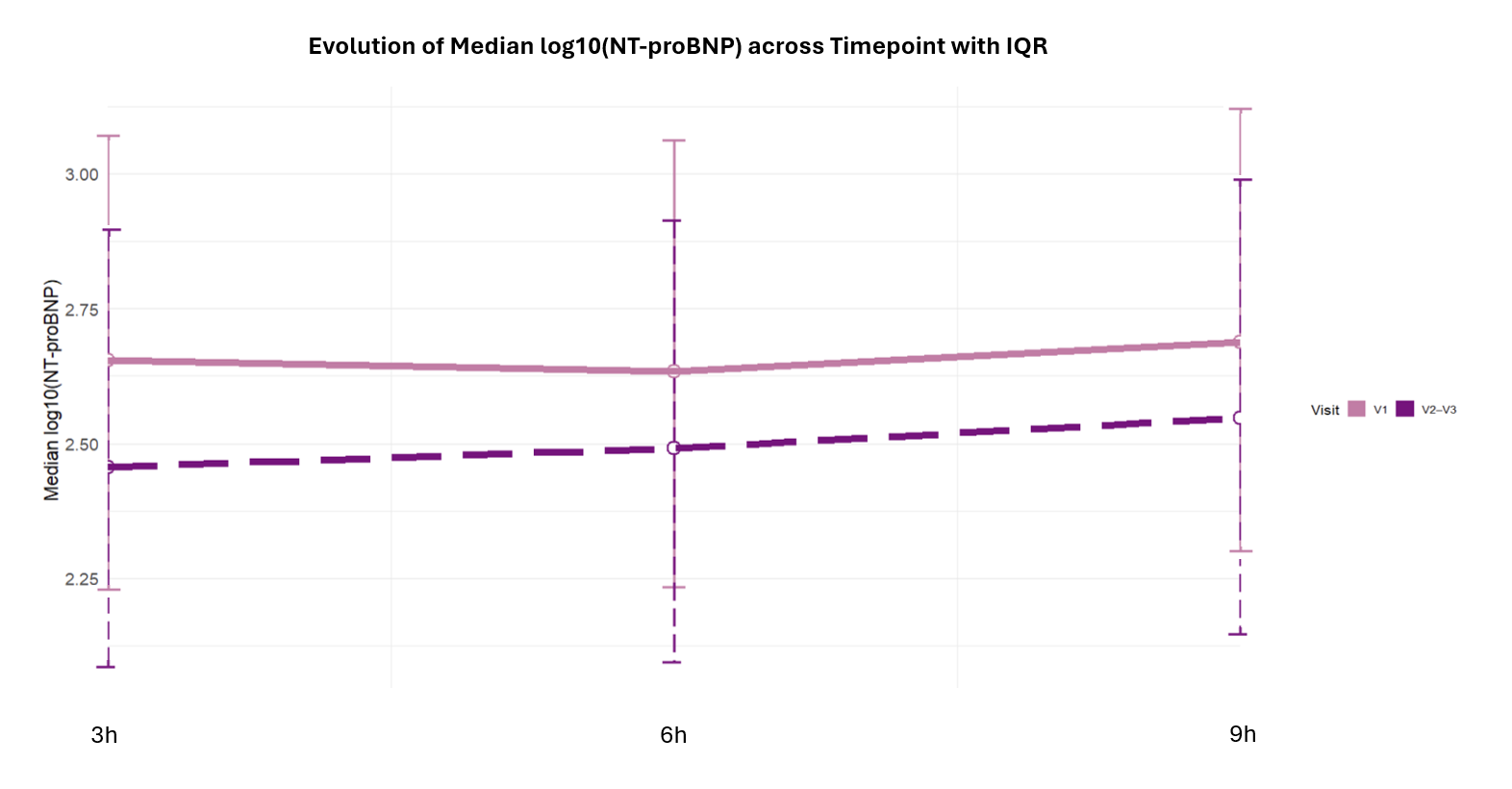
**

**Figure 2.**

**2a) Adjusted geometric mean BNP by timepoint (3, 6 and 9 hours): back-transformed EMMs, pooled over visits.** Points and lines show the back-transformed EMMs (geometric means) by timepoint, adjusted for visit and pooled across V1 and V2/3. Error bars show 95% confidence intervals (CI).

**
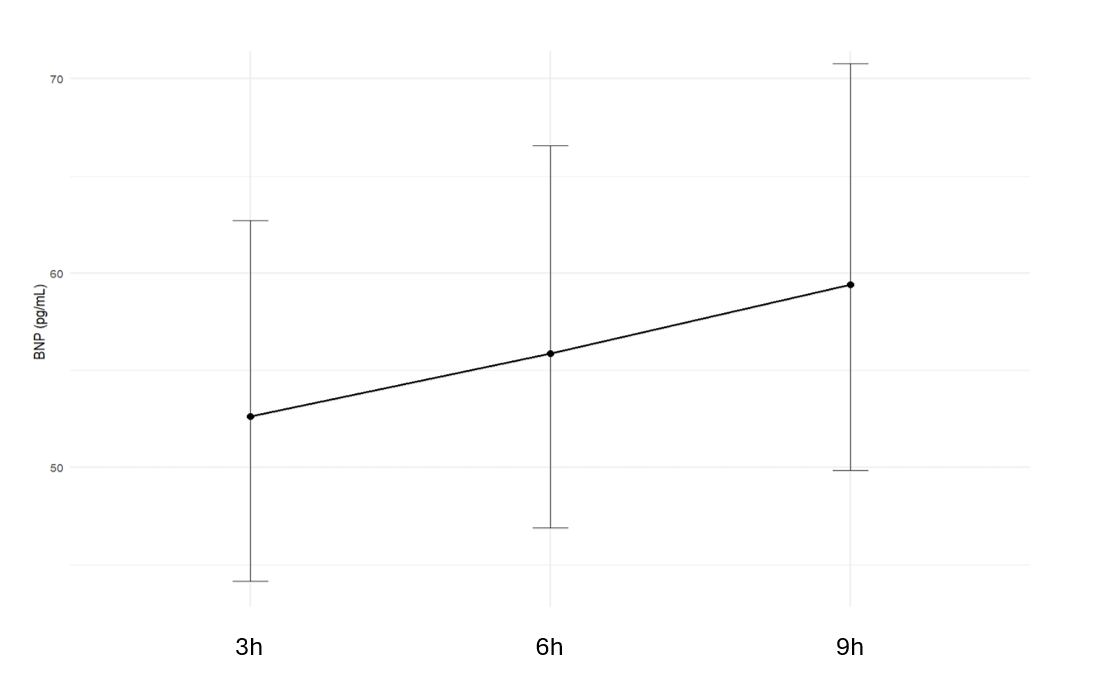
**

**2b) Adjusted geometric mean BNP by visit group (V1 vs V2/3): back-transformed estimated marginal means, pooled across timepoints.** Back-transformed estimated marginal means (reported as geometric means) for BNP by visit group (V1 vs V2/3), pooled across timepoints (3, 6 and 9 hours). Error bars show 95% confidence intervals.

**
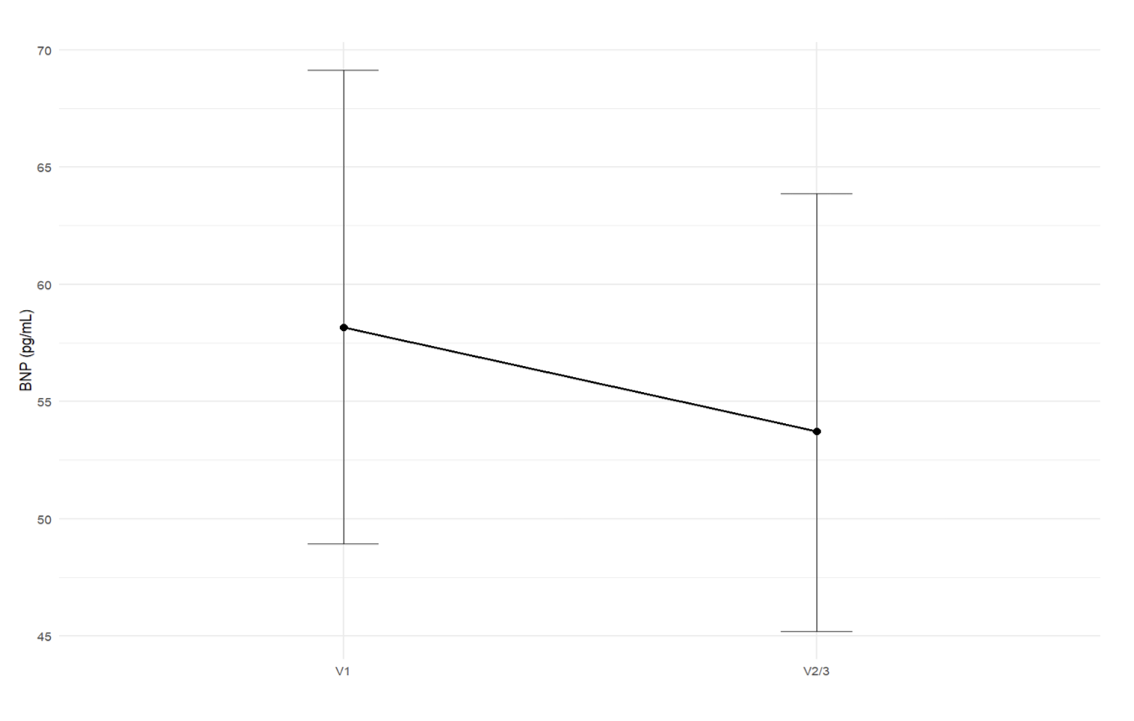
**

**Figure 3.**

**3a) Adjusted geometric mean NT-proBNP by timepoint (3, 6 and 9 hours): back-transformed EMMs, pooled over visits.** Points and lines show the back-transformed EMMs (geometric means) by timepoint, adjusted for visit and pooled across V1 and V2/3. Error bars show 95% confidence intervals (CI).

**
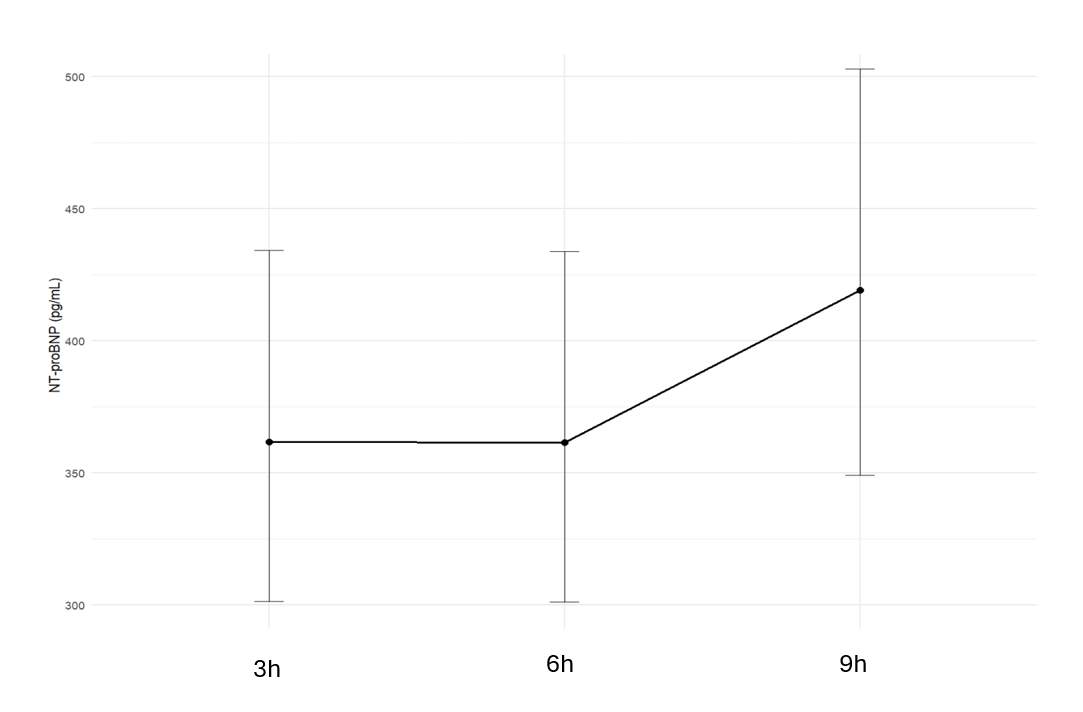
**

**3b) Adjusted geometric mean NT-proBNP by visit group (V1 vs V2/3): back-transformed estimated marginal means, pooled across timepoints.** Back-transformed estimated marginal means (reported as geometric means) for NT-proBNP by visit group (V1 vs V2/3), pooled across timepoints (3, 6 and 9 hours). Error bars show 95% confidence intervals.

**
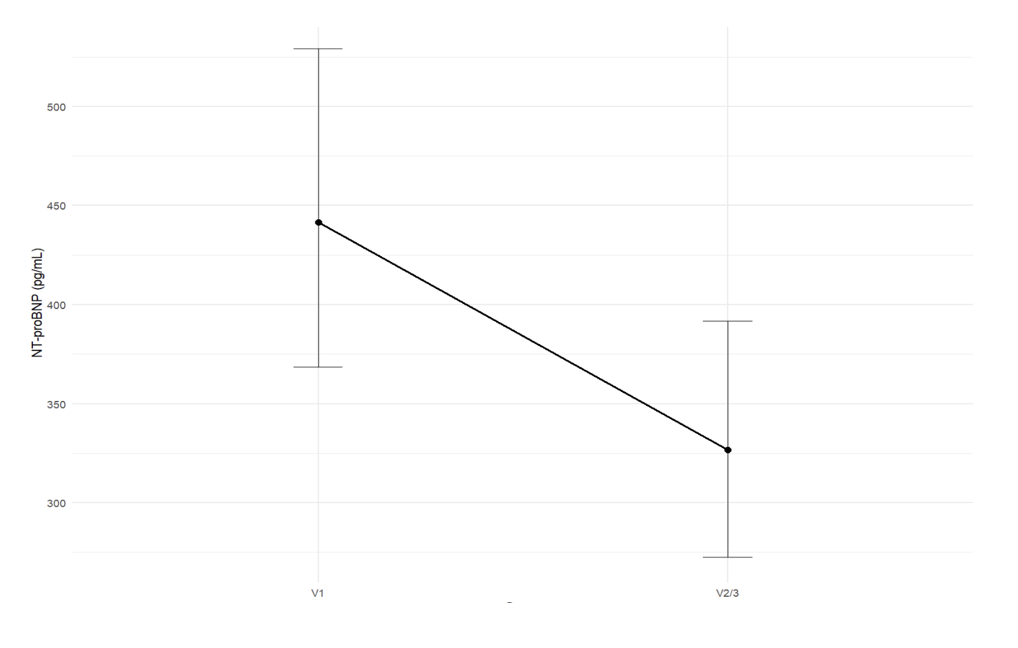
**

**Figure 4. Changes in BNP and NT-proBNP according to natriuretic response during the diuretic phase (H6–H9). Changes in log-transformed BNP (panel A) and NT-proBNP (panel B) between 6 and 9 hours, stratified by cumulative natriuretic response during the diuretic phase (H6–H9). Patients were classified as having a high or low natriuretic response based on the median cumulative natriuresis. Boxes represent median and interquartile range. No significant differences were observed between groups (all p=ns).**

**4a.**

**
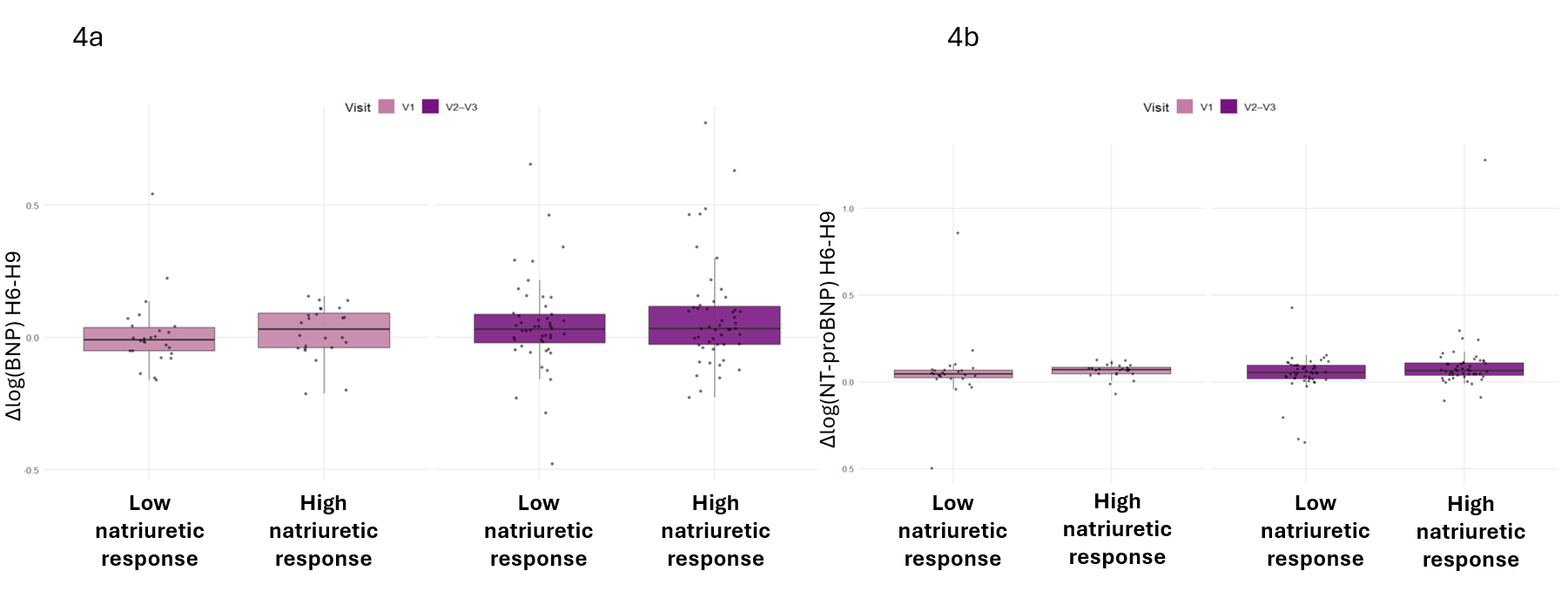
**

**4b.**

**
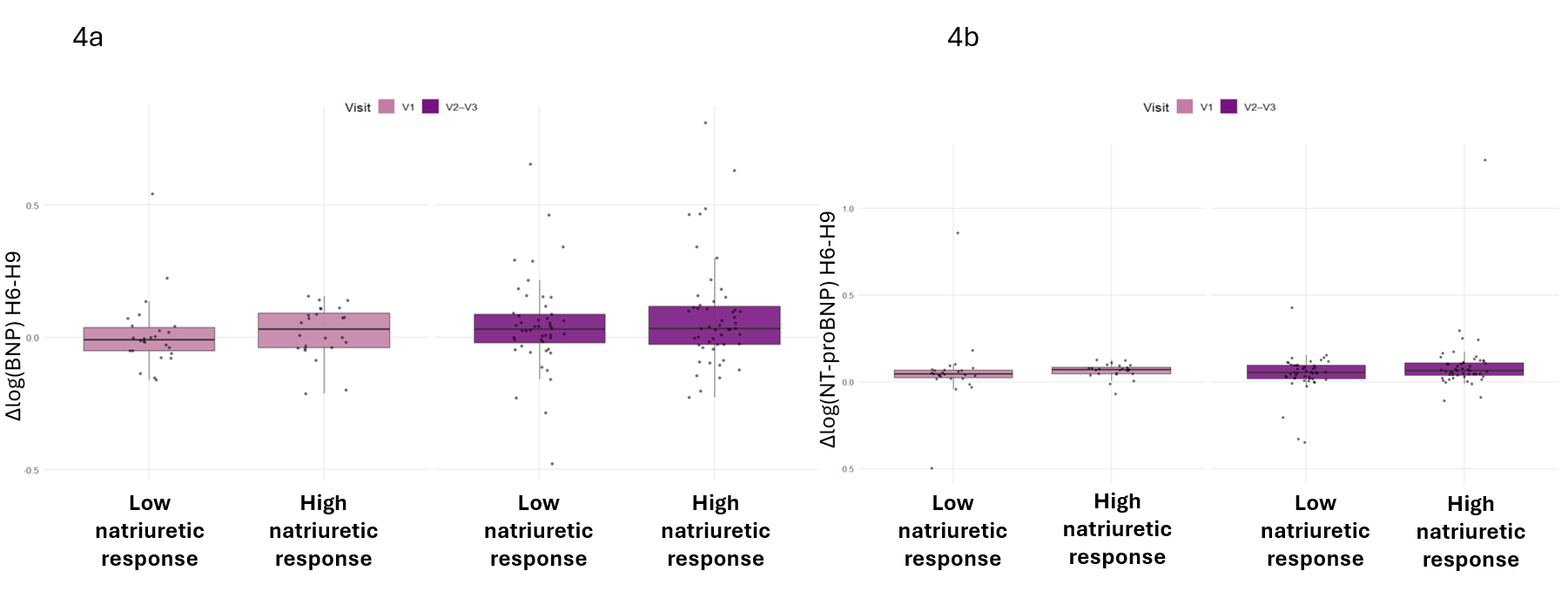
**
